# Shifts in the developmental rate of spadefoot toad larvae cause decreased complexity of post-metamorphic pigmentation patterns

**DOI:** 10.1038/s41598-020-76578-1

**Published:** 2020-11-12

**Authors:** Lee Hyeun-Ji, Miguel Ángel Rendón, Hans Christoph Liedtke, Ivan Gomez-Mestre

**Affiliations:** 1 Ecology, Evolution, and Development Group, Doñana Biological Station, Consejo Superior de Investigaciones Científicas, 41092 Seville, Spain; 2 Department of Wetland Ecology, Doñana Biological Station, Consejo Superior de Investigaciones Científicas, 41092 Seville, Spain

**Keywords:** Evolutionary ecology, Evolutionary developmental biology

## Abstract

Amphibian larvae are plastic organisms that can adjust their growth and developmental rates to local environmental conditions. The consequences of such developmental alterations have been studied in detail, both at the phenotypic and physiological levels. While largely unknown, it is of great importance to assess how developmental alterations affect the pigmentation pattern of the resulting metamorphs, because pigmentation is relevant for communication, mate choice, and camouflage and hence influences the overall fitness of the toads. Here we quantify the variation in several aspects of the pigmentation pattern of juvenile spadefoot toads experimentally induced to accelerate their larval development in response to decreased water level. It is known that induced developmental acceleration comes at the cost of reduced size at metamorphosis, higher metabolic rate, and increased oxidative stress. In this study, we show that spadefoot toads undergoing developmental acceleration metamorphosed with a less complex, more homogeneous, darker dorsal pattern consisting of continuous blotches, compared to the more contrasted pattern with segregated blotches and higher fractal dimension in normally developing individuals, and at a smaller size. We also observed a marked effect of population of origin in the complexity of the pigmentation pattern. Complexity of the post-metamorphic dorsal pigmentation could therefore be linked to pre-metamorphic larval growth and development.

## Introduction

The evolution of colour and pigmentation as a means of signalling is a common phenomenon across the tree of life^1^. This signalling is important for species recognition^2^, social interactions within species such as mate choice and intra-sexual competition, interactions between species such as aposematism, mimicry or invitations for pollination and seed dispersal, and for camouflage to avoid detection. Much empirical work has focused on pigmentation as honest signals indicating individual quality^3^. Classic examples of this are colour patches in birds that can convey honest information about parasite and pathogen resistance^4,5^. Yet fewer empirical studies have focused on describing features of animal pigmentation *pattern* (e.g. complexity, heterogeneity, lacunarity), and its relationship to body condition or stress endured^6^. This is especially relevant for camouflage, where patterning is just as important as the pigmentation itself^7–10^.

When colouration is not uniform, the arrangement of different coloured features can generally be defined as spots, stripes or polygons^11^. These three arrangements have evolved many times independently in the animal kingdom and play an important role in communication (e.g. egg spots in cichlids^12^, splotches in cuttlefish^13^, barred plumage in birds^14^) and crypsis (e.g. disruptive contrast^7^, outline- and surface disruption^8^, and counter shading^15^). This two- and sometimes three-dimensional attribute of colouration can vary not only between species, but also between individuals within species^16^ and often times serve as signal of an individual’s quality for mate choice. Both colour and its patterning are highly evolvable and the correct formation of patterning can have direct fitness consequences, especially in the context of camouflage^17,18^ and mimicry^19,20^.

In the same way that fluctuating asymmetry resulting from disruption to development can be used as a signal of individual quality, the correct formation of patterning is contingent on developmental homoeostasis^6^. Patterning has a strong genetic component^16,21^ although the formation of complex patterns can result from developmental alterations^11^, possibly influenced by environmental stressors^22^. Indeed, a higher pattern complexity in the black bib of red-legged partridges has been shown to be an indication of higher individual quality^23^. Likewise, ornament symmetry in little bustards^24^ and facial patterning of paper wasps^25^ also reflect the developmental conditions experienced.

While much of the research on colour patterning in vertebrates has focused on birds and teleost fish^1^, amphibians have been an important system in this topic as well^2,26–28^. A characteristic feature of many amphibians is their complex life cycle, which most typically consists of an aquatic larval stage and a terrestrial adult phase^29^. In fact, amphibian larvae and adults differ physiologically, anatomically, and ecologically^30^ to the extent that they are at times conceptually treated as separated units^31,32^. Nonetheless, multiple studies show that changes in the developmental trajectory of larvae have direct effects on adults^33–35^. This is particularly relevant for pigmentation, as the adult patterning forms during late stages of the larval development and metamorphosis^36^. Hence, if development of pigmentation is altered during this critical period, the adult phenotype will be directly affected. This has been shown in zebrafish, which undergo similar complex life cycles, where environmental stress at the larval stage affects adult pigmentation^37^. Amphibian larvae can decouple growth and differentiation to adjust their development to local environmental conditions^35,38^, constituting an ideal system to test for consequences of plastic alterations of development on pigmentation. Understanding the evolution of colour patterning in adult frogs, regardless of its function in signalling or camouflage, is therefore contingent on understanding how larval development affects the correct formation of patterns.

Here we test whether the dorsal pigmentation pattern of juvenile spadefoot toads (*Pelobates cultripes*) is affected by developmental alterations during the larval phase. Adults are semi-fossorial and nocturnal, typically living in sandy substrate where they can bury themselves easily. Their dorsal colour patterning can be described as darkly mottled with uneven blotches on a light uniform background and likely serves camouflage by background matching. Its larvae can grow for extended periods of time, reaching large sizes at metamorphosis under benign conditions, but can accelerate development and precipitate metamorphosis if at risk of desiccation from pond drying^39^. Developmental acceleration is in part achieved through increased corticosterone levels and metabolic rate and although critical for evading drying ponds, it comes at the cost of smaller size at metamorphosis, higher oxidative stress, reduced immunocompetence and allometric changes in body shape^39–41^. To test whether post-metamorphic pigmentation patterning is impacted by environmentally induced acceleration of pre-metamorphic larval development in spadefoot toads, we reared individualized tadpoles from multiple egg clutches and from four populations in standardized conditions, and experimentally induced accelerated development in half of them by reducing their water level. Upon metamorphosis, we compared body mass, snout-to-vent length, and dorsal patterning between accelerated and non-accelerated individuals. Patterning was quantified using digital imaging to estimate parameters describing different aspects of the texture and complexity of pigmentation.

## Results

### Effects of water level on growth and development

Experimentally decreasing water level led tadpoles to accelerate their development by 21% on average (F_1,150_ = 24.8, *p* < 0.001), reducing the age when Gosner stage 46 was reached from 171 ± 55 days to 132 ± 44 days. Larvae subjected to reduced water levels metamorphosed at lower body mass and shorter body length: body mass was decreased from 1.84 ± 0.97 g to 1.24 ± 0.31 g, representing 34% decrease (F_1,150_ = 86.36, *p* < 0.001), and snout-to-vent length was decreased from 26.62 ± 12.13 mm to 23.39 ± 4.79 mm, representing a 14% decrease (F_1,150_ = 101.4, *p* < 0.001).

### Principal component analysis of pigmentation pattern characteristics

**Figure 1:**
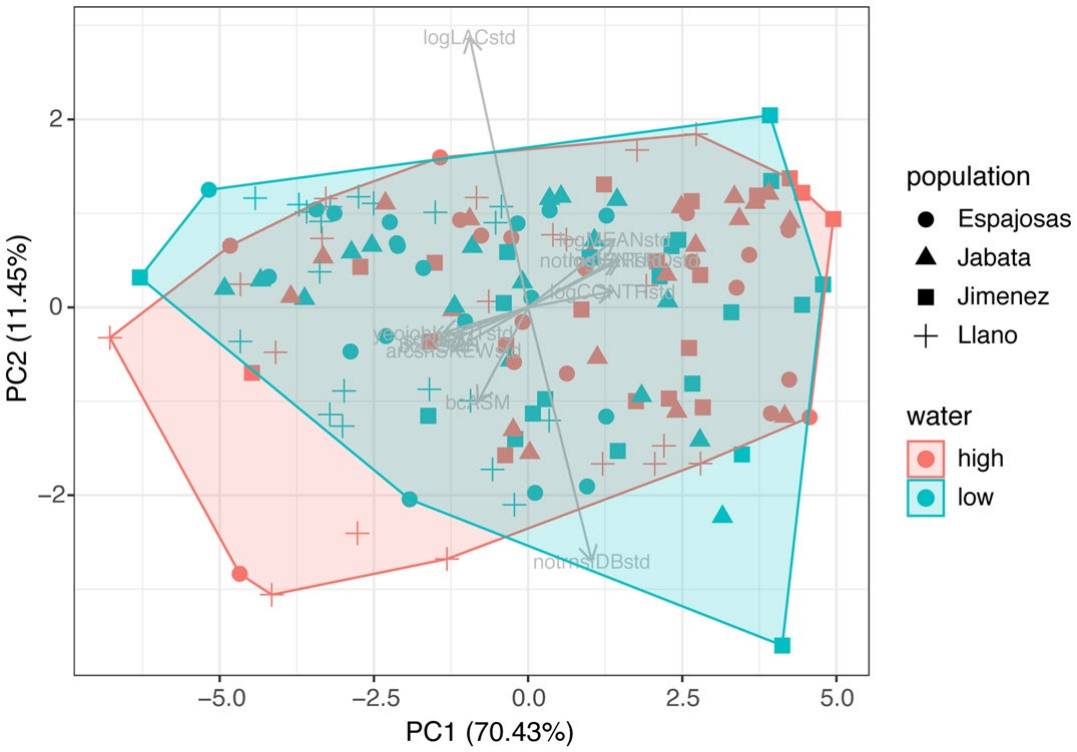
Principal component analysis: Most variables that loaded heavily on the first principal component (*entropy*, *mean gray value, gray value variance,* and *contrast* pointing to opposite directions than *kurtosis*, *correlation* and *inverse difference moment, while all loaded at a similar magnitude*) explained 70.43% of the variation. The second principal component explained 11.45% of the variation and was heavily loaded by *fractal dimension* and *lacunarity*.

Eleven variables have been calculated to describe 3 features: frequency distribution of grey scales (*mean, variance, skew,* and *kurtosis*), homogeneity (*angular second moment, inverse difference moment, entropy, contrast,* and *correlation*), and complexity (*lacunarity* and *fractal dimension*). We observed substantial variation in pigmentation patterns in the metamorphosed toads emerging from our experiment. A principal component analysis showed that most of the calculated pattern descriptors loaded considerably on the first principal component, with *entropy*, *mean, variance, and contrast* of the grayscale distribution of the pattern showing similar magnitudes but opposite directions to *kurtosis*, *correlation* and *inverse difference moment* (S1 in Supplementary material). *Fractal dimension* and *lacunarity* loaded heavily on the second principal component while loading in opposite directions, *angular second moment* loaded on the third principal component, and *skewness* on the fourth component. The numerous variables showing similar loadings on PC1 indicated high collinearity among variables, which we further visualized using the *ggpair* function of the R package GGally^42^ (S2 in Supplementary material). Principal components 1, 2, and 3 together explained over 89% of the variance in the pigmentation patterns across toads (S1 in Supplementary material). The PC scores largely overlapped across populations and across treatments (Fig. 1).

### Effects of developmental acceleration, population and sibship on pigmentation pattern

**Figure 2:**
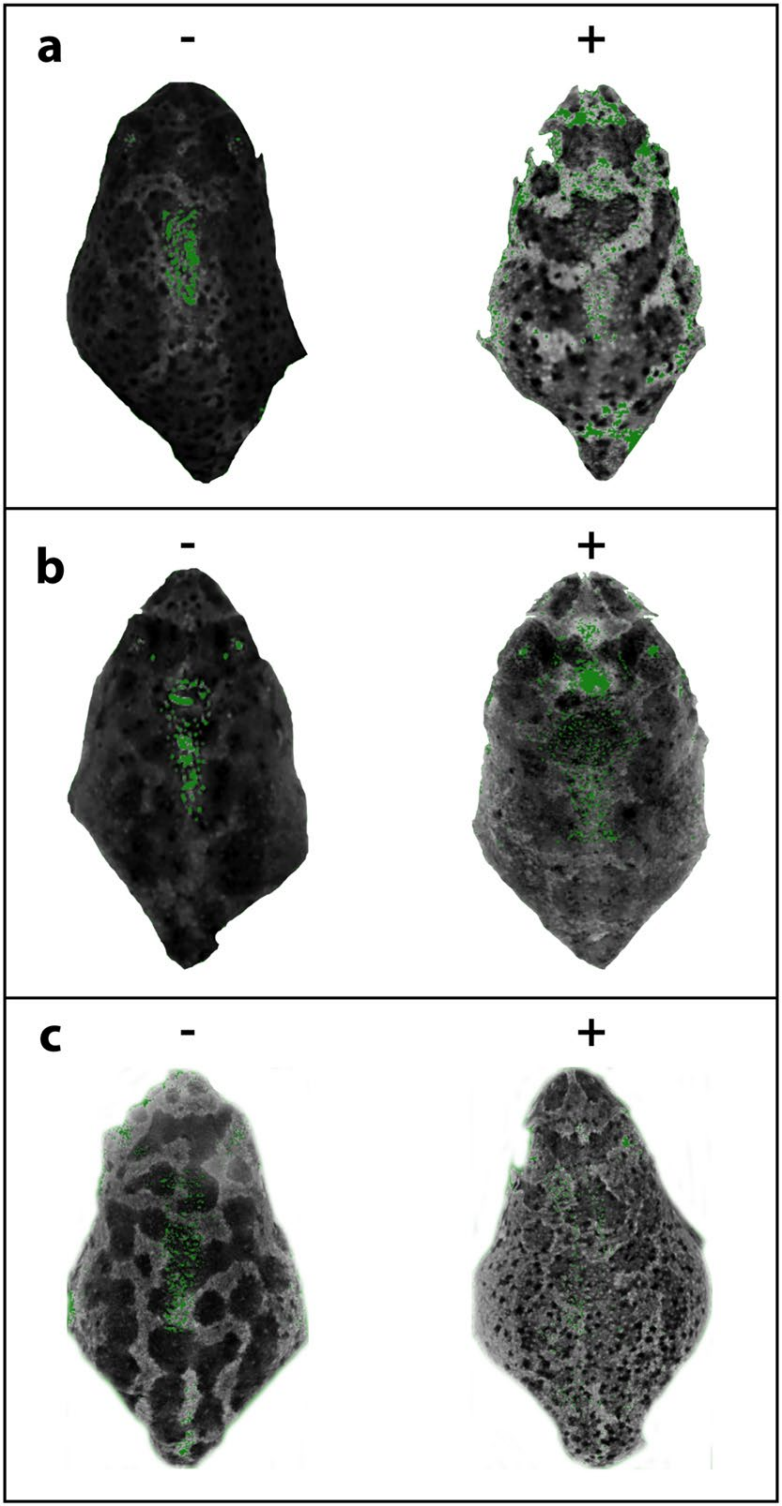
Photographic representation of dorsal pigmentation pattern differences as described by the three most informative patterning statistics (**a**) mean grey value, (**b**) fractal dimension and (**c**) angular second moment. The left panel (−) shows exemplary individuals with low extremes and the right pane (+) shows exemplary individuals with high extremes for each descriptor. When raised under environmentally stressful (i.e. low water level treatment) conditions, toads metamorphose with an overall darker dorsal pattern (i.e. lower mean grey value), with decreased heterogeneity (i.e. lower fractal dimension) and with a more homogeneous dorsal pattern (i.e. higher angular second moment).

We used Bayesian hierarchical linear models to test for the effects of experimentally decreasing water level on multivariate pigmentation pattern, specifically on the *mean* of the grayscale distribution, *angular second moment,* and *fractal dimension* chosen based on their high loading on different principal components and low collinearity among each other (Fig. 2; see “Material and methods” section for more detail). Our model comparisons based on measures of the prediction accuracy by expected pointwise predictive density (ELPD) showed that the model including sibship nested within population as random (group) effect was the better fit than the one excluding it: the predictive ability was improved by adding the random effect according to both the loo (ΔELPD: fit1 = 0.0, fit0 = − 42.8; SE: fit1 = 0.0, fit0 = 15.5) and waic criteria (ΔELPD: fit1 = 0.0, fit0 = − 43.3; SE: fit1 = 0.0, fit0 = 15.6), where fit1 is the model including random effects and fit0 is the model excluding random effects, respectively. Therefore, it could be inferred that sibship and population effect explained a considerable fraction of the variance of each of these variables and we included *population* and *sibship* (clutch) as random effects in the subsequent models. Since aspects of the pigmentation pattern seemed to be attributable to sibship, we estimated broad sense heritability (*H*^2^) of these three variables (Table 1) using the *sommer*^43^ package in R.

**Table 1:** Estimates of the genetic variance component (*V*_G_) and broad sense heritability (H^2^) for the three main pigmentation traits studied, along with their associated standard errors (SE).

Pigmentation trait	*V*_G_		*H*^2^	
	Estimate	SE	Estimate	SE
Mean grayscale	0.274	0.135	0.30	0.10
Fractal dimensión	0.147	0.086	0.15	0.08
Angular second moment	0.182	0.095	0.18	0.08

The Bayesian generalized coefficients of the first model including only water treatment as fixed effect (fit1) showed that the experimental treatment (i.e. water level) explained a considerable fraction of the variance of the variables, while more so in *mean* and *angular second moment* than in *fractal dimension* (mean: *R*^2^ = 0.29, fractal dimensions: *R*^2^ = 0.17, angular second moment: *R*^2^ = 0.23). Such proportion of variance explained increased when larval period was added as an additional fixed effect (fit2), and again, *mean* and *angular second moment* were better explained than fractal dimension (mean: *R*^2^ = 0.36, fractal dimensions: *R*^2^ = 0.20, angular second moment: *R*^2^ = 0.31). Consistently, including body mass at metamorphosis as additional fixed effect improved the model fit (fit3), and mean and angular second moment were better explained than fractal dimension (mean: *R*^2^ = 0.37, fractal dimension: *R*^2^ = 0.19, angular second moment: *R*^2^ = 0.31). In all models, 100% of the observations had a lower pareto *k* value than 0.7, indicating optimal effective sample size.

Our model comparisons based on measures of the prediction accuracy by expected pointwise predictive density (ELPD) showed that the model including both experimental treatment (i.e. water level) and larval period was the best fit (fit2). This model had the highest predictive ability according to both the loo (ΔELPD: fit2 = 0.0, fit3 = − 1.1, fit1 = − 5.3; SE: fit2 = 0.0, fit3 = 3.6, fit1 = 5.2) and waic criteria (ΔELPD: fit2 = 0.0, fit3 = − 1.0, fit1 = − 4.7; SE: fit2 = 0.0, fit3 = 3.5, fit1 = 5.6).

**Figure 3:**
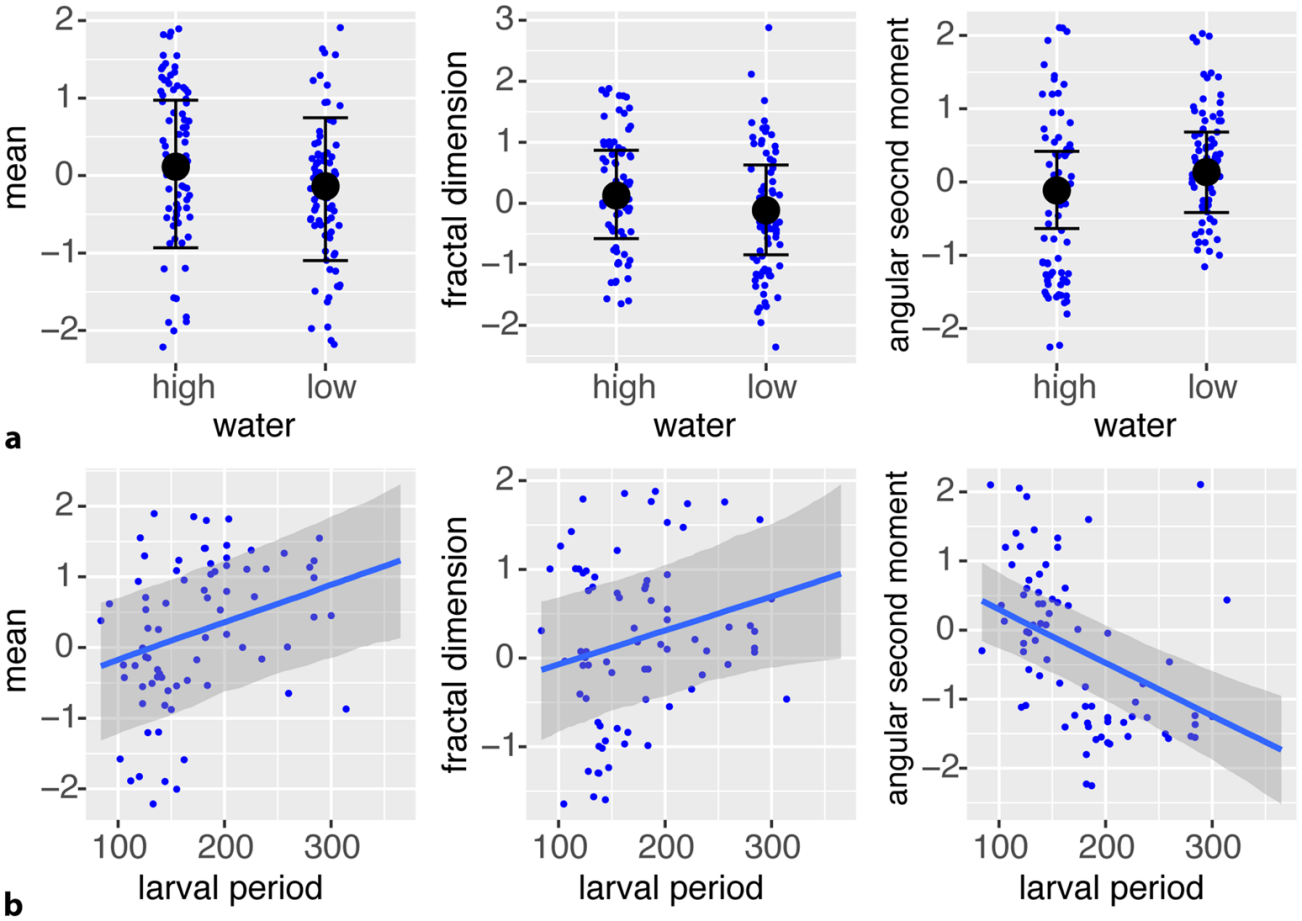
Conditional effects of the best fitting Bayesian model including water level treatment and larval period as fixed effects showed that decreased water levels led to lower greyscale mean and fractal dimension and higher angular second moment values. Note that the big black dots visualize the model estimates (predicted values of the response), the upper and lower bounds stand for the uncertainty intervals of the response, and the blue dots represent the original data points. Furthermore, the duration of the larval period was positively correlated with mean and fractal dimension while negatively with angular second moment.

The conditional effects of this best fitting model (Fig. 3) showed that decreased water levels led to lower mean of the grayscale distribution, lower fractal dimension and higher angular second moment. Similarly, the duration of the larval period was positively associated with mean grayscale and fractal dimension and negatively with angular second moment.

## Discussion

The dorsal pigmentation pattern of *P. cultripes* metamorphs varied in texture and complexity across populations and sibships, but also in response to altered developmental rate. The among-sibship and among-population variation is intriguing because it suggests a heritable component. Our estimated broad-sense heritability for brightness, homogeneity and complexity aspects of the pigmentation patterns ranged between 0.15–0.30, although our full sib design prevented us from disentangling additive genetic from non-additive and maternal components^44^ and should only be considered a general indication of a hereditary component of the pattern.

As expected, spadefoot toad tadpoles accelerated their development in response to reduced water level, resulting in tadpoles metamorphosing earlier but at a smaller size. Interestingly, this developmental acceleration of the larvae also caused alterations in different aspects of the dorsal pigmentation patterns of the emerging toads. Toads that had accelerated their larval development (i.e. experienced stressful conditions) showed a less contrasted and more homogenous pattern (less random in its intensity, as interpreted based on low fractal dimensions and high angular second moment values; Figs. 2, 3) compared to control toads (i.e. benign conditions), with the blotches being darker and more highly connected amongst each other. Toads induced to accelerate their development also showed a lower complexity of their pigmentation pattern and lower fractal dimension, or “gappiness”^6^.

Developmental acceleration in response to pond drying is known to take a considerable physiological toll on amphibian larvae^41,45^. In order to achieve such acceleration, spadefoot toads increase the corticosterone and thyroid hormone secretion and their metabolic rate^39,46^. The formation of the adult pigmentation pattern in the epidermis of amphibians is induced during metamorphosis by the melanocyte-stimulating hormone (MSH), in interaction with thyroid hormone^47^, to the extent that hypophysectomy causes bleaching of tadpole skin^48^. For instance, in oriental fire-bellied toad tadpoles (*Bombina orientalis*), experimental inhibition of thyroid hormone led to a marked reduction in the size of melanophores on their dorsal skin^26^. We thus hypothesize that enhanced pituitary activity in response to perceived risk of predation may trigger over-melanization of tadpole skin as they approach metamorphosis. Sexual hormones are also known to affect the development of pigmentation pattern in amphibians, and disruption of these hormones cause profound changes to the pigmentation of post-metamorphic amphibians^49–51^, although it is still unclear how induced developmental acceleration may affect the secretion of these hormones. Pigments such as carotenoids are known to prevent oxidative stress^52^ and enhance the coloration of metamorphosing frogs^53^, and their availability is reduced in birds when raised under stressful conditions^52,54^. In amphibians, however, a direct link between pigmentation and oxidative stress is not yet clearly established as not enough studies have explored amphibian pigmentation in the context of environmental stress experienced during their larval period.

Rather, past studies have focused on pigmentation as aposematism^55,56^ or camouflage^57^. Interestingly, deformed individuals originating from stressful conditions showed larger pigment spots despite being smaller in size in comparison to individuals originating from benign conditions in northern leopard frogs (*Rana pipiens*)^58^. Newts have been shown to be capable of background matching^59,60^, and newts that expressed more pigments have been shown to have a higher metabolic rate, suggesting a metabolic cost entailed in pigment expression^60^, but it is still unknown whether such increased metabolism has carry-over consequences.

While we find that *P. cultripes* metamorphs originating from stressful conditions (i.e. accelerated development) show darker and more continuous blotches, at this stage it is hard to tease apart whether this was to compensate for smaller size resulting from limited time of growth or rather a side effect of developmental acceleration and insufficient time for growth. Although variation in coloration can often play a role in thermoregulation, UV protection, predator avoidance, or sexual signaling^27,61,62^ we do not yet know whether variation in the pigmentation pattern of this burrowing, nocturnal species with aquatic acoustic communication is of any adaptive value. Adaptive coloration in amphibians remains a vast field to be explored, as for instance, subterranean caecilians have evolved bright coloration^63^. *Pelobates cultripes* is mostly an amphibian of nocturnal habits (although males may stay by the clutch during daytime on the first day post-oviposition) and its dorsal pigmentation likely serves as camouflage rather than communication^64^. However, whether the observed variation in pigmentation and complexity across populations is an evolved response to different levels of predation pressure and whether developmental alterations of such patterns increase their risk of predation remain open questions. For now, our finding that tadpoles induced to accelerate their development emerged with a more homogeneous and darker pigmentation pattern (Figs. 2, 3) suggests two hypotheses. First, the development of lighter, well-contrasted, complex dorsal patterns in normally developing individuals may be disrupted due to physiological stress, indicating deterioration of body condition that carries over to the juvenile phase. Second, the darker and more homogenous pigmentation of accelerating individuals may simply reflect heterokairic effects (i.e. environmentally induced shifts in the timing of developmental events^65,66^), wherein skin development did not proceed at the same pace as that of other organs in accelerating individuals. Individuals subject to developmental acceleration may not have had enough time and/or resources to allocate for the optimal expression of pigmentation, which is what might have led to their darker and less complex patterns.

Amphibians show remarkable variation in pigmentation patterns, both within and among species. Here we show that patterns can be altered due to plastic alterations of development. Future studies would have to determine whether the texture and complexity of the pattern are heritable, the relative importance of parental effects, whether it is static or changes ontogenetically, and whether it shows geographic variation or phylogenetic signals.

## Material and methods

### Study animals and preparation of dorsal pigmentation photos

We collected portions of five egg clutches of four *P. cultripes* populations from southern Spain (Huelva province) and reared them in climatic chambers of Estación Biológica de Doñana, CSIC (Seville). Upon reaching the free-feeding stage, 14 tadpoles per sibship (clutch) were individualized in 4 L round plastic containers. They were kept in identical conditions (20 °C, carbon filtered dechlorinated tap water) and fed a mixture of ground up rabbit chow (alfalfa) and spinach. At Gosner stage 35 (i.e. stage at which digits are identifiable on the hindlimb bud^65^), we dropped the water volume to 650 mL in half the containers to simulate risk of pond drying. Gosner stage 35 is the optimal developmental stage at which spadefoot toad larvae are able to perform the most acceleration in comparison to other developmental stages^67^. The experimental procedures were approved by CSIC’s IACUC committee (authorization #560-2017), and all experiments were performed in accordance with relevant guidelines and regulations.

### Image acquisition and preprocessing

We recorded the duration of the larval period of each toad, marked by the completion of metamorphosis (full tail resorption, Gosner stage 46)^68^ at which stage we also measured snout-to-vent length (to the nearest 0.1 mm) and body mass (to the nearest 0.1 mg). A dorsal photograph of each toad placed over laminated grid paper was taken with a Nikon D50 camera. Since photos were taken from slightly different distances, images were downscaled to the same resolution (13.2 px/mm) by bicubic interpolation. We focused our analysis of pigmentation patterns on the torso and head (except eyeballs), excluding the limbs to avoid noise from variation in posture. Regions of Interest (ROI) on the toad’s dorsal surface were manually outlined.

The resulting ROI images were transformed to grayscale 8-bit images (256 intensity values). Intensity values of images were obtained from the image histogram, in which the reflectance of each pixel ranged from 0 to 255. Uneven illumination was corrected using a pseudo-flat field correction tool (blurring radius: 40 pixels). Contrast was enhanced by a histrogram stretching to a full 8-bit range of gray (0.1% saturated pixels). The unsharp masking filter tool of imageJ (weight value of 0.5 and blur radius of 1.0) was applied to improve sharpness. Due to artificial light sources, wet dorsal skin of toads showed specular reflection, which may potentially have resulted in errors in image analyses. We therefore applied threshold criteria to determine areas affected by specular reflection, and removed them from the ROIs (intensity values > 190).

### Extraction of textural features

Image texture can be defined as the spatial arrangement of intensities^69,70^. Skin texture was characterized by measuring the likelihood of observing an intensity value at randomly selected pixels in the images (i.e. first-order statistics) and the spatial relationships between them (i.e. second-order statistics)^69,70^. From the frequency distribution of gray scales in the images, the following first-order statistics were calculated: mean ($\mu$), variance (${\sigma }^{2}$), skewness (*S*), and kurtosis (*K*). Second-order textural features were extracted using the Gray Level Co-occurrence Matrix (GLCM)^70–73^. GLCM is a matrix representation of an image in which the gray tone of each pixel is quantized to a set (*G*) of *N*_*θ*_ levels. Information on texture is specified by the matrix of relative frequencies *P*_*(i,j|d,θ)*_ in which two neighboring pixels separated by distance d and angle *θ* occur on an image, one with gray tone *i* and another with *j*^71^. GLCMs of irregular ROIs were calculated from a distance between pixel (*d*) = 1 at four directions (*θ*) = 0°, 45°, 90°, and 135°, and then averaged for each image. In order to characterize the textural features of the dorsal images of the toads, we obtained the following five GLCM measurements:

i) Angular second moment (*ASM*): measure of homogeneity, high values indicate very few dominant gray-scale transitions (i.e. homogeneous images).
  $$ASM = \sum_{{i = 0}}^{{G - 1}} \sum_{{j = 0}}^{{G - 1}} P\left\{ {\left( {i,j} \right)} \right\}^{2}$$
ii) Contrast (*CON*): measure of local intensity variation between a continuous set of pixels.
  $$CON = \mathop \sum \limits_{n = 0}^{G - 1} n^{2} \left\{ {\mathop \sum \limits_{i = 0}^{G - 1} \mathop \sum \limits_{j = 0}^{G - 1} P(i,j)} \right\},{ }\left| {i - j} \right| = n$$
iii) Inverse difference moment (*IDM*): weighted by the inverse of the contrast (${\text{i}} \ne {\text{j}}$) which is influenced by homogeneity, high values indicate homogeneous textures:
  $$IDM = \mathop \sum \limits_{i = 0}^{G - 1} \mathop \sum \limits_{j = 0}^{G - 1} \frac{{P\left( {i,j} \right)}}{{1 + \left( {i - j} \right)^{2} }}$$
iv) Entropy (*ENT*): statistical measure of information content estimating the randomness of intensity distribution of textures (i.e. Images presenting low entropy are homogeneous):
  $$ENT = - \mathop \sum \limits_{i = 0}^{G - 1} \mathop \sum \limits_{j = 0}^{G - 1} P\left( {i,j} \right) \times \log \left( {P\left( {i,j} \right)} \right)$$
v) Correlation (*COR*): measure of linear dependency between gray scales on neighboring pixels at the specified positions. High correlation values indicate images with regions presenting similar intensity:
  $$COR = \mathop \sum \limits_{i = 0}^{G - 1} \mathop \sum \limits_{j = 0}^{G - 1} \frac{{\left\{ {i \times j} \right\} \times P\left( {i,j} \right) - \left\{ {\mu_{i} \times \mu_{j} } \right\}}}{{\sigma_{i} \times \sigma_{j} }}$$

($\mu$ stands for the mean and $\sigma$ for the standard deviation of brightness)

The plug-in tool GLCM Texture v.0.008^74,75^ of the software ImageJ was used.

### Spatial heterogeneity (fractal dimension and lacunarity) analysis

Fractal dimension of gray-scale images^76^ was measured on ROIs applying differential box-counting methods using the plug-in FracLac in ImageJ^77^ A series of boxes (samples) of decreasing scale (*ε*) were projected over a gray-scale image with an intensity *I*_(*i,j*)_ scaled to a range of 0 to 255 for each pixel (*i,j*). For each box, the difference between the maximum and minimum values of pixel intensity (δ*I*_*i,j,ε*_) was calculated and the slope of the log–log regression between box size and the sum of all the intensity differences (*Iε* = ∑ [1 + δ*I*_*i,j,ε*_]) was used to calculate the gray fractal dimension (${D}_{\mathrm{B}})$. Fractal dimension constitutes a measure of spatial complexity^78–80^:

$$D_{B} = \mathop {\lim }\limits_{{n \to \infty }} \ln (I_{\varepsilon } )/\ln (1/\varepsilon )$$

Lacunarity (λ) is a measure of spatial heterogeneity that complements fractal dimension; whereas *D*_B_ measures *how much* space is filled, lacunarity describes the spatial size of gaps and measures *how* space is filled^81^ Thus, a fractal with large and heterogeneous gaps has high lacunarity values and vice versa:

$$\lambda = CV_{{\varepsilon ,g}}^{2} = (\sigma /\mu )^{2}$$

where *σ* stands for the standard deviation, *μ* for the mean difference in intensity for pixels per box at the scale *ε* and orientation *g*.

### Statistical analyses

Variables were normalized using the *bestNormalizer*^82^ package in R (version 3.6.2). Fractal dimension (*D*_B_) and entropy required no transformation, whereas mean (*μ*), variance (*σ*^*2*^), contrast, and lacunarity (*λ*) were log-transformed. A Box Cox transformation was applied to angular second moment (*ASM*) and correlation (*COR*), and a square root to inverse difference moment (*IDM*). We then standardized each variable to a mean of 0 and standard deviation of 1. We then performed a principal component analysis (PCA, R version 3.6.2.) on the transformed variables, using factor loadings to assess the relative contribution of each variable to the variation observed in each of the principal components. Some of these variables were complementary to each other and showed high collinearity. Thus, multiple variables provided similar information regarding the frequency distribution of grey scales (*mean*, *variance*, *skewness*, *kurtosis*) whereas others described homogeneity in the pattern (*ASM*, *IDM*, *entropy*, *contrast*, *correlation*) and yet others spatial complexity (*lacunarity*, *fractal dimension*). We visualized the pairwise relationship among these variables using the *ggpair* function of the R package GGally^42^, and selected three variables of interest with low collinearity among them and loading on different principal components: *mean*, *angular second moment,* and *fractal dimension*. We then fitted multivariate Bayesian hierarchical linear models on these three variables using the R package *brms*^83^. We began with comparing model performance including and excluding sibship and population as random (group) effects, and included those in further analyses as models performed better with random effects. We compared model performance of fitting either on i) only water treatment, ii) water treatment and larval period, or iii) water treatment and body mass upon metamorphosis as fixed (population) effects, including sibship nested within population as random (group) effect for each model. All three response variables in the models were fitted within a gaussian distribution with default prior settings, with 152 observations (i.e. metamorphs) and 4 group levels (i.e. populations) for each model. Two sampling chains ran for 2000 iterations with a warm-up period of 1000 iterations for each model, hence yielding 2000 samples for each parameter. As diagnostic checks, chain convergence, autocorrelations and posterior predictive distributions were visually inspected and effective sample sizes calculated. We also checked for low pareto k heritage (< 0.7) for each observation within each model. To explore how much variation in the response variables were explained by our models, we employed a Bayesian generalization of the *R*^2^ coefficient. Finally, the best model fit (i.e. the model with the highest predictive accuracy based on the expected log pointwise predictive density [ELPD]) was evaluated across models by estimating the out-of-sample predictive fit using the leave-one-out cross-validation (loo) and widely applicable information criterion (waic) criteria.

## Supplementary information

Supplementary Table 1.

Supplementary Information.

## Data Availability

Data are archived in an institutional public repository (Digital CSIC: https://hdl.handle.net/10261/219661).
