# Supplementary material for: Shifts in the developmental rate of spadefoot toad larvae cause decreased complexity of post-metamorphic pigmentation patterns
Source: Sci Rep. 2020 Nov 12;10:19624. doi: 10.1038/s41598-020-76578-1 (PMC7665075; doi:10.1038/s41598-020-76578-1)
Supplement: Supplementary file 1 — Supplementary Table 1. [file 41598_2020_76578_MOESM1_ESM.pdf]

**Shifts in the developmental rate of spadefoot toad larvae cause decreased complexity of post-metamorphic pigmentation patterns**

Lee Hyeun-Ji[1,2], Miguel Ángel Rendon[2], Hans Christoph Liedtke[1,2], and Ivan Gomez-Mestre[1,2]*

[1]Ecology, Evolution, and Development Group, [2]Department of Wetland Ecology, Doñana Biological Station, Consejo Superior de Investigaciones Científicas, 41092 Seville, Spain

*Corresponding author: igmestre@ebd.csic.es

Table 1. Factor loadings of Principal Component Analysis (PCA)

| | PC1 | PC2 | PC3 | PC4 |
|---|---|---|---|---|
| entropy | -0.347* | -0.118 | 0.048 | 0.158 |
| mean | -0.329* | -0.169 | -0.148 | -0.267 |
| variance | -0.332* | -0.118 | 0.311 | 0.038 |
| skewness | 0.282 | 0.107 | 0.258 | 0.621* |
| kurtosis | 0.323* | 0.064 | 0.031 | 0.373 |
| angular second momentum | 0.191 | 0.236 | 0.752* | -0.462 |
| contrast | -0.321* | -0.040 | 0.350 | 0.287 |
| correlation | 0.337* | 0.095 | -0.310 | -0.097 |
| inverse difference moment | 0.340* | 0.079 | 0.030 | -0.262 |
| fractal dimensions | -0.242 | 0.633* | -0.088 | 0.047 |
| lacunarity | 0.222 | -0.674 * | 0.138 | -0.012 |
| Eigenvalues | 7,75 | 1,26 | 0,85 | 0,72 |
| Percentage of variation explained (%) | 70,43 | 11,45 | 7,71 | 6,59 |
