# Supplementary material for: Shifts in the developmental rate of spadefoot toad larvae cause decreased complexity of post-metamorphic pigmentation patterns
Source: Sci Rep. 2020 Nov 12;10:19624. doi: 10.1038/s41598-020-76578-1 (PMC7665075; doi:10.1038/s41598-020-76578-1)
Supplement: Supplementary file 2 — Supplementary Information. [file 41598_2020_76578_MOESM2_ESM.pdf]

Shifts in the developmental rate of spadefoot toad larvae cause decreased complexity of post-metamorphic pigmentation patterns

Lee Hyeun-Ji[1,2], Miguel Ángel Rendon[2], Hans Christoph Liedtke[1,2], and Ivan Gomez-Mestre[1,2]*

[1]Ecology, Evolution, and Development Group, [2]Department of Wetland Ecology, Doñana Biological Station, Consejo Superior de Investigaciones Científicas, 41092 Seville, Spain

*Corresponding author: igmestre@ebd.csic.es

Supplementary material 2. Pairwise relationship among the study variables quantifying texture and complexity of the pigmentation pattern of juvenile spadefoot toads (*Pelobates cultripes*) using the ggpair function of the R package GGally.

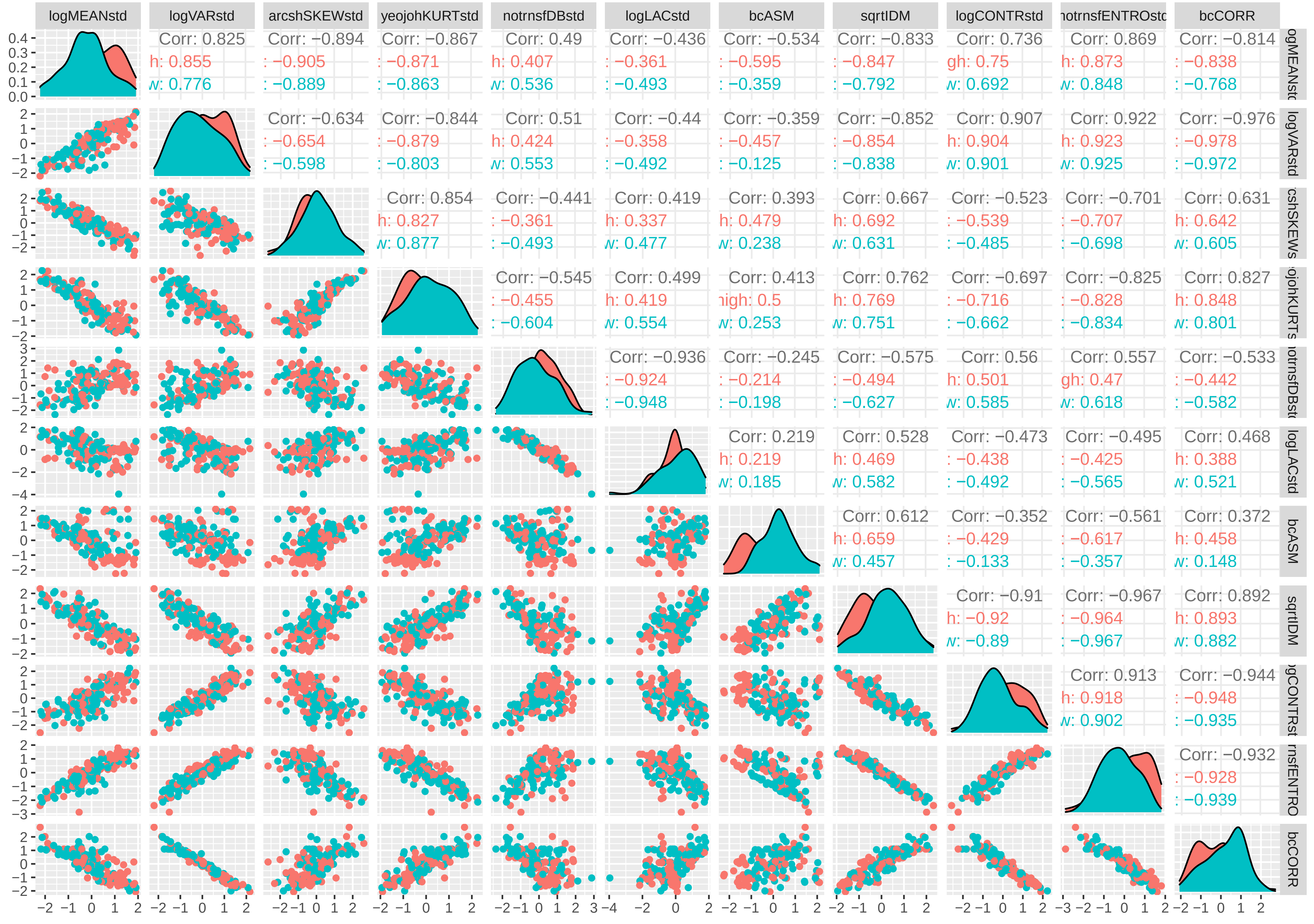
logMEANstd
logVARstd
arcshSKEWstd
yeojohKURTstd
notrnsfDBstd
logLACstd
bcASM
sqrtIDM
logCONTRstd
notrnsfENTROstc
bcCORR
Corr: 0.825
h: 0.855
w: 0.776
Corr: −0.894
: −0.905
: −0.889
Corr: −0.867
: −0.871
: −0.863
Corr: 0.49
h: 0.407
w: 0.536
Corr: −0.436
: −0.361
: −0.493
Corr: −0.534
: −0.595
: −0.359
Corr: −0.833
: −0.847
: −0.792
Corr: 0.736
gh: 0.75
w: 0.692
Corr: 0.869
h: 0.873
w: 0.848
Corr: −0.814
: −0.838
: −0.768
Corr: −0.634
: −0.654
: −0.598
Corr: −0.844
: −0.879
: −0.803
Corr: 0.51
h: 0.424
w: 0.553
Corr: −0.44
: −0.358
: −0.492
Corr: −0.359
: −0.457
: −0.125
Corr: −0.852
: −0.854
: −0.838
Corr: 0.907
h: 0.904
w: 0.901
Corr: 0.922
h: 0.923
w: 0.925
Corr: −0.976
: −0.978
: −0.972
Corr: 0.854
h: 0.827
w: 0.877
Corr: −0.441
: −0.361
: −0.493
Corr: 0.419
h: 0.337
w: 0.477
Corr: 0.393
h: 0.479
w: 0.238
Corr: 0.667
h: 0.692
w: 0.631
Corr: −0.523
: −0.539
: −0.485
Corr: −0.701
: −0.707
: −0.698
Corr: 0.631
h: 0.642
w: 0.605
Corr: −0.545
: −0.455
: −0.604
Corr: 0.499
h: 0.419
w: 0.554
Corr: 0.413
igh: 0.5
w: 0.253
Corr: 0.762
h: 0.769
w: 0.751
Corr: −0.697
: −0.716
: −0.662
Corr: −0.825
: −0.828
: −0.834
Corr: 0.827
h: 0.848
w: 0.801
Corr: −0.936
: −0.924
: −0.948
Corr: −0.245
: −0.214
: −0.198
Corr: −0.575
: −0.494
: −0.627
Corr: 0.56
h: 0.501
w: 0.585
Corr: 0.557
gh: 0.47
w: 0.618
Corr: −0.533
: −0.442
: −0.582
Corr: 0.219
h: 0.219
w: 0.185
Corr: 0.528
h: 0.469
w: 0.582
Corr: −0.473
: −0.438
: −0.492
Corr: −0.495
: −0.425
: −0.565
Corr: 0.468
h: 0.388
w: 0.521
Corr: 0.612
h: 0.659
w: 0.457
Corr: −0.352
: −0.429
: −0.133
Corr: −0.561
: −0.617
: −0.357
Corr: 0.372
h: 0.458
w: 0.148
Corr: −0.91
h: −0.92
w: −0.89
Corr: −0.967
: −0.964
: −0.967
Corr: 0.892
h: 0.893
w: 0.882
Corr: 0.913
h: 0.918
w: 0.902
Corr: −0.944
: −0.948
: −0.935
Corr: −0.932
: −0.928
: −0.939
ogMEANstc
logVARstd
cshSKEWs
ojohKURTs
otrnsfDBstc
logLACstd
bcASM
sqrtIDM
gCONTRst
rnsfENTRO
bcCORR
